# Legal restrictions on foreign institutional investors in a large, emerging economy: A comprehensive dataset

**DOI:** 10.1016/j.dib.2019.104819

**Published:** 2019-11-16

**Authors:** Radhika Pandey, Rajeswari Sengupta, Aatmin Shah, Bhargavi Zaveri

**Affiliations:** NIPFP, IGIDR, L&L Partners, FRG-IGIDR, India

**Keywords:** Foreign institutional investors, Capital control actions, Debt, Equity, Derivatives, Financial regulation

## Abstract

The dataset presented in this article contains information on imposition or relaxation of legal restrictions on foreign investment, by the authorities in a large, emerging economy- India. These restrictions are referred to as capital controls because they act as controls on the capital account of an economy. Legal instruments such as regulations, circulars and notifications published on the websites of the relevant regulatory authorities, have been used as the source of the data. In particular, the dataset discerns information from these legal instruments to identify whether the instrument tightens or eases capital controls on investment by foreign institutions in different asset classes such as debt, equity and derivatives.

**Table:** Specifications Table

Subject area	*Law and Economics*
More specific subject area	*Finance, Foreign investment, Capital account liberalisation*
Type of data	*Tables, graphs*
How data was acquired	*Hand collection of legal restrictions from legal instruments published on the websites of regulatory agencies. Microsoft Excel Version 16.29.1 was used to generate the graphs.*
Data format	*Raw and analysed*
Experimental factors	*The data-set classifies capital control measures into different categories and scores these measures depending on whether they relax or restrict investment by foreign institutional investors in the Indian economy.*
Experimental features	*Foreign investors are critical for financing investment in emerging economies such as India where domestic saving falls short of the investment requirements.*
Data source location	*Websites of regulatory agencies*
Data accessibility	*Raw data is available here:*http://ifrogs.org/releases/Pandeyetal2019_Legalrestrictions_ForeigninstitutionalInvestors.html

**Table:** 

**Value of the Data** • The data described in this article allows us to build a time-series of capital control measures that have been imposed in India. This can be used to understand the extent to which India's capital account has gradually opened up since the economic liberalisation reforms of the mid 1990s, over a period of more than 20 years. • The data can help construct indices for measuring the *de-jure* capital account openness of India with respect to foreign portfolio investment. The data can also be used to analyse the circumstances in which these instruments were introduced to evaluate their impact on outcomes such as foreign investment inflows into India, currency volatility, inflation and cost of capital in the economy. • The capital controls dataset and related statistics presented in this article will give policy makers an overview of the evolution of legal restrictions on foreign portfolio investment in Indiaover time, the frequency of changes that have been brought about and their impact on policy objectives. • The data presented in this article will allow finance practitioners and foreign investors to understand the current state of capital account openness in India which in turn may help them undertake investment decisions.

## 1 Data

**Fig. 1:**
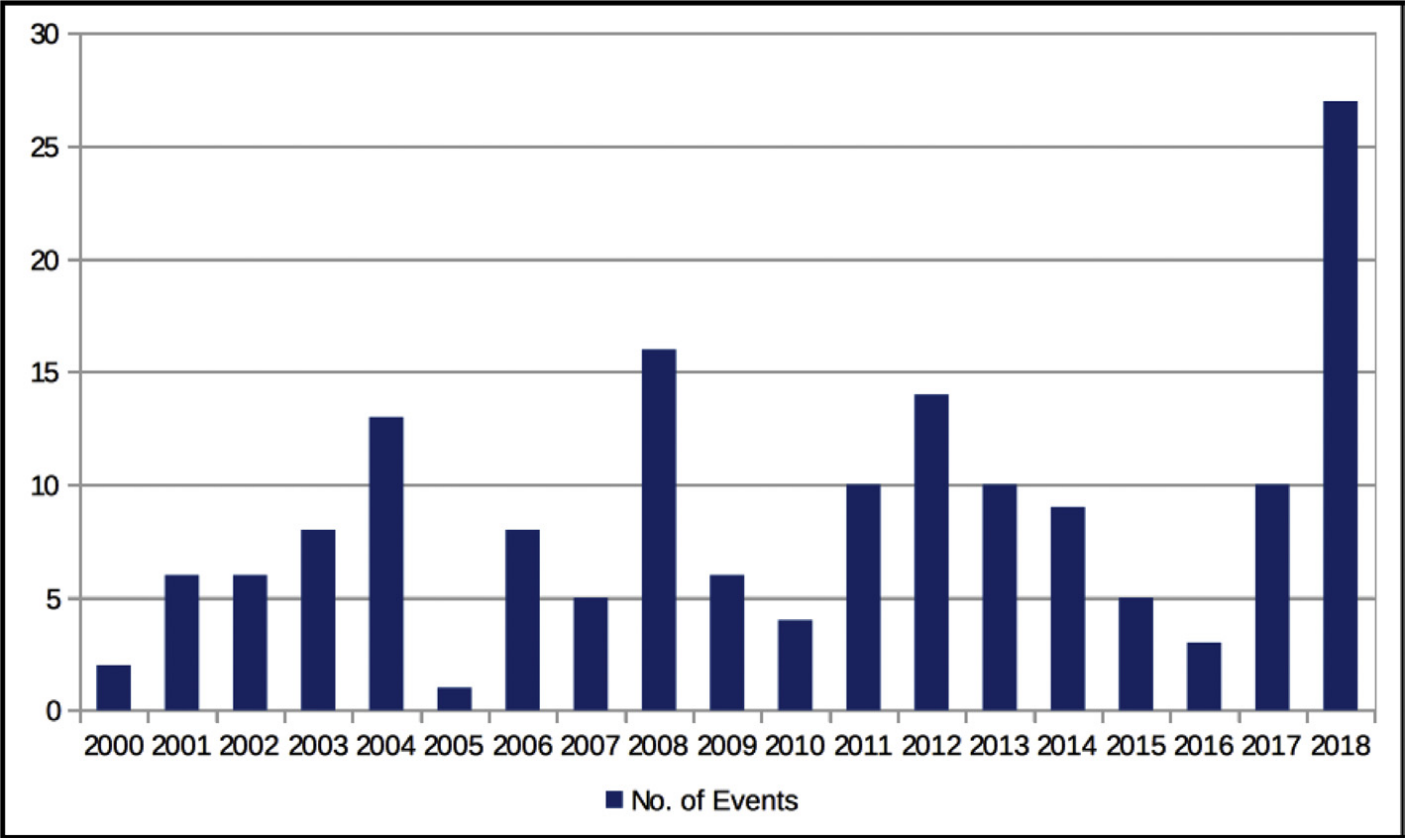
Annual distribution of capital control events.

**Fig. 2:**
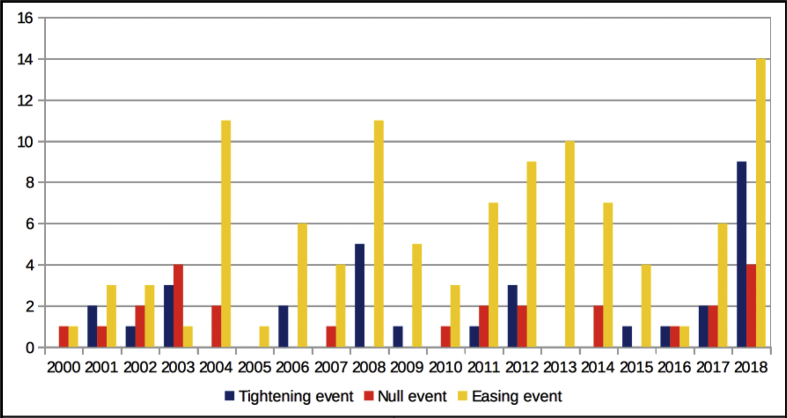
Annual distribution of easing, tightening and null capital control events.

**Fig. 3:**
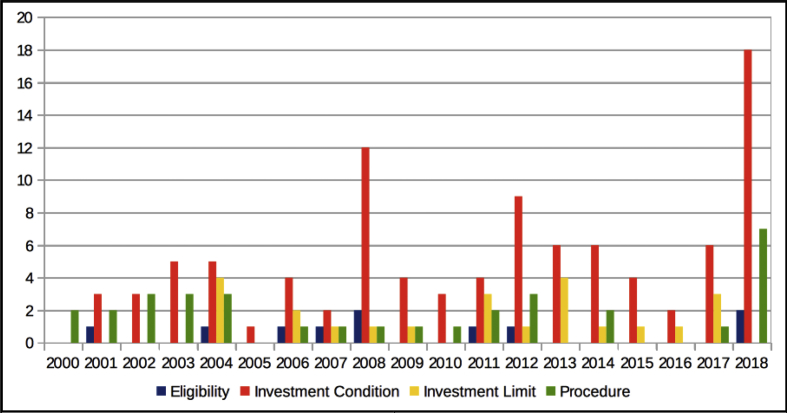
Categories of capital control events.

**Fig. 4:**
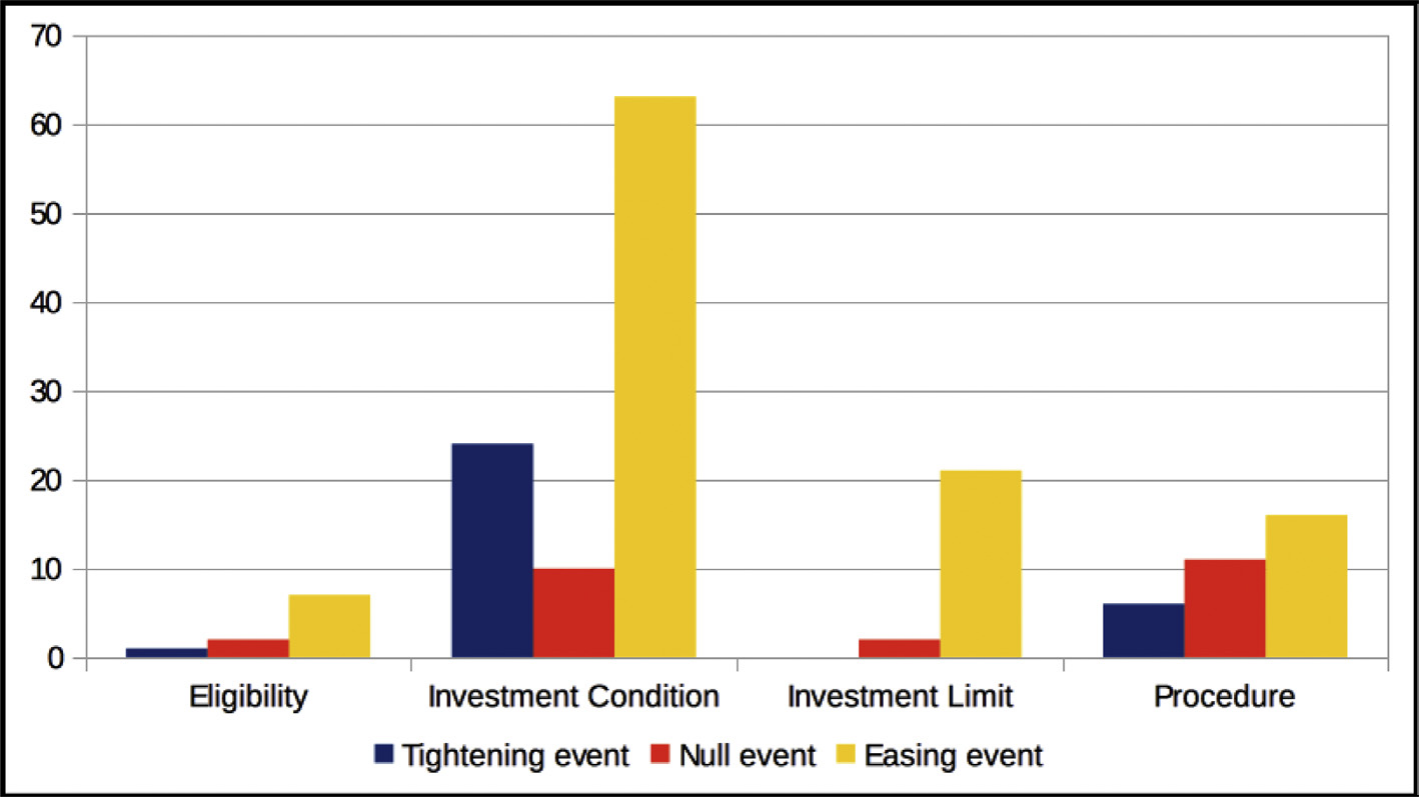
Easing and tightening of different types of capital controls.

**Fig. 5:**
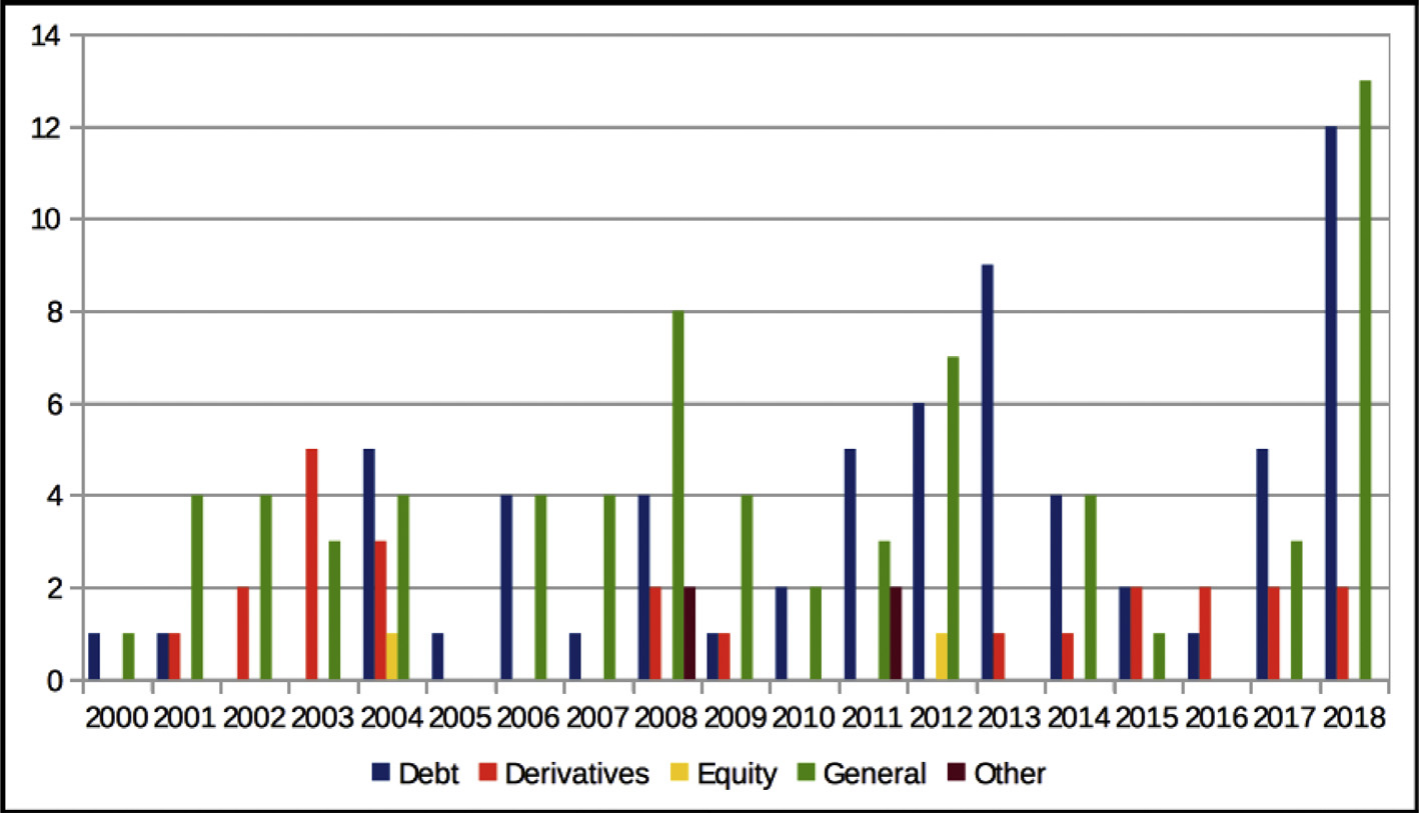
Annual distribution of capital controls in different asset classes.

**Fig. 6:**
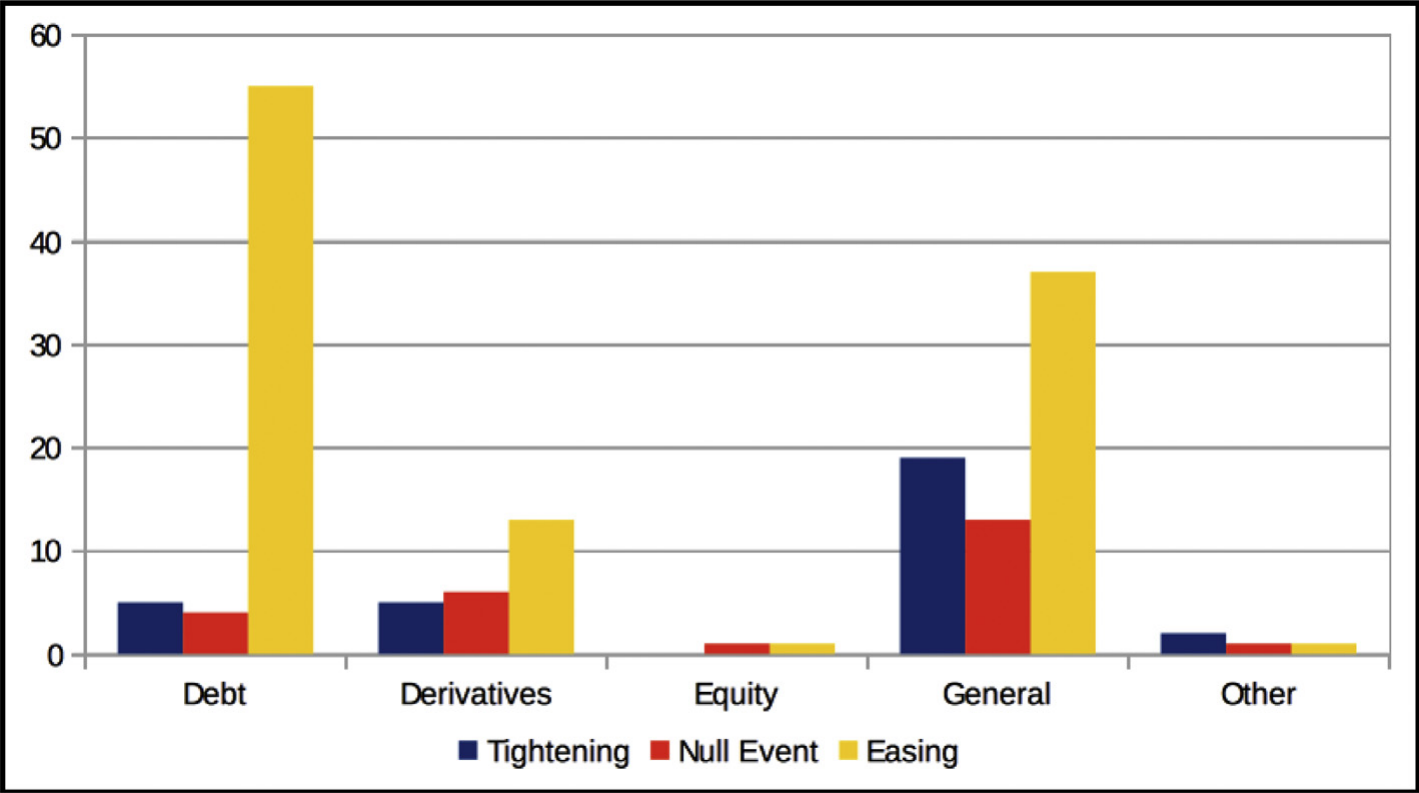
Annual distribution of easing and tightening events across asset classes.

The dataset quantifies the legal regulations applicable to foreign portfolio investors interested in investing in the Indian financial markets.^1^ These regulations are referred to as capital controls because they act as a control on the capital account of an economy. The capital account is a summary of inflows and outflows of foreign investment to and from the host country, which in this case is India. This data records the easing and tightening of capital controls on foreign portfolio investors. Fig. 1 shows the number of capital control events by year. Fig. 2 shows the number of easing and tightening capital control events by year. Fig. 3 shows the year-wise distribution of capital control events by the type of capital control involved. Fig. 4 shows the easing and tightening of the various types of capital controls. Fig. 5 describes these capital controls by the asset classes that they affect. Fig. 6 shows the number of easing and tightening of capital controls across the different asset classes.^2^

The traditional approach to measuring capital controls has relied on cross-country de-jure measures such as the Chinn-Ito index and the Schindler index [2,3]. These measure the level of capital account openness using the summary classification tables published by the International Monetary Fund in the Annual Report on Exchange Arrangements and Exchange Restrictions (AREAER). While these measures are useful for cross-country comparisons, they have limited utility for understanding a country-specific legal framework dealing with capital controls. This is especially true for a country like India, which has an elaborate legal and administrative framework governing capital controls. The cross-country indices of capital controls detect a movement towards capital account openness only when a specific category of controls is dismantled. In India, while the structure of controls is intact, many restrictions have been administratively or procedurally eased leading to greater access for foreign investors. As an outcome, these indices assign a score to India that has not changed from 1970 to 2017.

To address these difficulties, the recent literature has shifted focus from level of capital controls to the precise measurement of capital control actions (CCAs). This paper is part of that emerging strand of literature. As an example, Pandey, Pasricha, Patnaik, and Shah (2016) [1] analyse legal documents to construct a dataset on restrictions on foreign currency borrowings by Indian firms. Foreign currency borrowing by firms (referred to as External Commercial Borrowings, ECB) is subject to a complex framework of capital controls on each aspect of borrowing, such as ceiling on the interest rate that can be paid, caps on the magnitude of borrowing, the uses that the borrowed amount can be put into etc. The authors construct a fine-tuned dataset tracking the easing and tightening of controls on all aspects of borrowings. Similarly, Forbes, Fratzscher, and Straub (2015) [4] analyse the motivations for imposing capital controls by constructing a dataset that tracks increases and decreases in controls on capital inflows, controls on capital outflows, and macro-prudential measures at a weekly frequency for 60 countries from 2009 through 2011.

The dataset was built by hand collecting qualitative information on the entire gamut of capital controls that were either imposed or relaxed by the concerned regulatory authorities in India with respect to foreign portfolio investment (henceforth, FPI) in India. Foreign portfolio investors are institutional investors. The concerned regulatory authorities include either the central bank of India, the Reserve Bank of India (RBI), or the securities regulator, the Securities and Exchange Board of India (SEBI). Our data spans a period of 18 years commencing on 1stJanuary, 2000 and ending on December 31, 2018. Our sample period begins in the year of operationalisation of the Foreign Exchange Management Act, 1999 (FEMA), which is the Indian law that governs foreign investment in India.

During this period, the total number of legal instruments issued with regard to FPI capital controls was 112. Separate instruments issued by the RBI and SEBI which have the same effect on capital controls are counted only once. Often, a single instrument makes multiple interventions in relation to capital controls. Each such intervention is referred to as a capital control event. We exclude interventions for which a legally binding source cannot be traced. Once all the changes are considered as separate events, the total number of capital control events is 151. On this basis, on average, India has faced roughly 8 or 9 capital control events annually during the period of this data.

Fig. 1 depicts the capital control events by year. In this span of 18 years, there was at least one capital control event each year. The year 2018 saw the maximum number of capital control events and the years 2000, 2005 and 2009 saw the least number of such events.

Capital control actions can be of two types—easing and tightening. In the rest of the paper, we refer to them as FPI easing events and FPI tightening events, respectively. FPI easing events denote events that have the effect of relaxation of existing controls or any action that makes it easier for foreign investors to invest in the host country. Conversely, FPI tightening events denote events that have the effect of increasing the capital controls or any actions that make it harder for foreign investors to invest in the host country. We find that for the full period of the dataset, the easing events are substantially higher in number at 99, compared to the tightening events which were 27 in number. For all the years, except 2003 and 2006, the number of easing events is higher than the number of tightening events.

Fig. 2 shows the annual distribution of easing and tightening events. The events that are (i) neither easing nor tightening or (ii) partially easing and partially tightening are classified, as null events. The maximum number of FPI easing events took place in 2018 (14 in number) followed by 2008 (11 in number) and 2013 (10 in number). The maximum number of FPI tightening events also took place in 2018 (9 in number) followed by 2008 (4 in number). 25 events are classified as null events.

We next consider two types of classifications of every capital control action: one based on the intended end-objective of the capital control action and the other based on the kind of assets to which the capital control action would apply. For each of these classifications, we further divide the events into easing and tightening events.

In the first classification scheme, we divide the capital controls into four categories depending on their intended end objective. These categories are:

• Eligibility: This category refers to capital control actions that decide the kind of foreign investors who might be eligible or ineligible to invest in the Indian financial markets.
• Investment condition: This category refers to capital control actions that govern the conditions of the investments that are undertaken by foreign investors. As an example, in 2004, FPIs were allowed to issue offshore derivative instruments against underlying securities held by them in the Indian stock exchange.
• Investment limit: These are capital control actions that deal with monetary limits up to which investments are permitted by FPIs.
• Procedure: The law on capital controls prescribes an elaborate administrative procedure that foreign investors need to follow in order to invest in the Indian debt and equity markets. For example, a change in the procedure for registration of an FPI with regulatory authorities in India will be classified in this category.

Fig. 3 depicts the FPI capital control actions classified into the above mentioned four categories during the sample period. We find that about 60% of the capital control actions during the period of the dataset relate to investment conditions, and 20% are ‘procedure’ related changes. The remaining 20% of the capital control actions relate to eligibility criteria or investment limits. The year 2018 witnessed the highest number of capital control actions in relation to investment conditions (18 in number), followed by 2008, and 2012.

In Fig. 4, we depict the number of easing and tightening events across the four categories. Of the FPI easing events during this period, more than half pertained to ‘investment conditions’ and the next largest chunk pertained to ‘investment limits’. The highest number of FPI tightening events was with respect to ‘investment conditions’. Thus, statistics show that majority of the capital control actions during the period from 2000 to 2018 have been in the domain of ‘investment conditions'.

In the second classification scheme, we split the capital control events into four main asset classes, namely-Debt, Derivatives, Equity and General. This classification helps understand which kind of assets witnessed the most capital control actions over the last two decades, as far as foreign institutional investment is concerned. “Debt” refers to investment in both corporate and government bonds. “Derivatives” include products such as equity futures and options, commodity derivatives etc.^3^ “Equity” refers to investment in the stocks and shares of Indian companies. Finally, those capital control changes that do not relate to changes in the asset class of “debt”, “equity” or “derivatives” but relate to, for example easing/tightening of procedures across all asset classes or easing/tightening of eligibility of FPIs across all asset classes, are grouped under the category of “general”.

We also create another category called “other” to track changes in asset classes other than “debt”, “equity” and “derivatives”. For example, FPI investments in Mutual Funds and Collective Investment Schemes (CIS) would be captured under the “others” category. As shown in Fig. 5, the “General” category saw the highest number of capital control actions (69) followed by “Debt” (64) and “Derivatives” (24). “Equity” (2) saw the least. The residual category of “Others” contained 4 capital control actions.

In Fig. 6, we plot the number of FPI easing events vs. FPI tightening events across the various asset classes. We find that the category “Debt” saw the highest number of capital controls easing whereas the “General” category faced the maximum tightening of controls.

## 2 Experimental design, materials, and methods

Cross-border capital flows coming into India are governed by FEMA and the rules and regulations made under it. India is currently a partially capital account convertible economy. Hence, under the current design of the legal framework, all capital account transactions in India are prohibited unless explicitly permitted. The permissions are granted through a set of legal instruments issued primarily by the central bank (RBI) and also by the securities market regulator (SEBI). Restrictions differ according to the type of foreign investor, the type of asset class, the intended recipient of foreign capital, the end use of foreign capital, etc.

In this article, we hand-construct a new dataset about one class of capital controls, those that affect the investment into India by foreign portfolio investors (FPIs). Changes to capital controls are published by the RBI and SEBI in their circulars which are publicly available. We analysed the text of these circulars to construct our dataset on capital controls governing foreign portfolio investments. The dataset classifies each capital control change as “easing” or “tightening”. Easing events are marked as “+1” and tightening events are marked as “–1”. The changes that are ambiguous or those that primarily relate to procedural changes are marked as “0”.

Since the liberalisation of India's economy in the 1990s, parts of its capital account has been liberalized and FPIs have been allowed to invest in Indian markets since 1992. In the 1990s, FPI investments were governed by Government of India guidelines and permissions under Foreign Exchange Regulation Act, 1973 (FERA), which was the legal framework preceding FEMA. With the enactment of FEMA in 1999, the capital controls governing FPIs came under the regulatory purview of RBI. Hence, we track the changes in FPI investments post the enactment of FEMA capturing all capital control actions with respect to FPIs from the year 2000–2018.

## Conflict of Interest

The authors declare that they have no known competing financial interests or personal relationships that could have appeared to influence the work reported in this paper.

## Footnotes

1 The dataset can be accessed here: http://ifrogs.org/releases/Pandeyetal2019_Legalrestrictions_ForeigninstitutionalInvestors.html.

2 In all the graphs the y-axis shows the number of capital control events.

3 Derivatives are securities that derive their value from an underlying debt instrument, share, loan or any other form of security. Derivatives may also derive their value from the prices or index of prices of the underlying securities.
